# Shearing in a Biomimetic Apatite-Protein Composite: Molecular Dynamics of Slip Zone Formation, Plastic Flow and Backcreep Mechanisms

**DOI:** 10.1371/journal.pone.0093309

**Published:** 2014-04-01

**Authors:** Dirk Zahn, Erik Bitzek

**Affiliations:** 1 Lehrstuhl für Theoretische Chemie/Computer Chemie Centrum, Friedrich-Alexander Universität Erlangen-Nürnberg, Erlangen, Germany; 2 Department of Materials Science and Engineering, Friedrich-Alexander Universität Erlangen-Nürnberg, Erlangen, Germany; Instituto de Tecnologica Química e Biológica, UNL, Portugal

## Abstract

We report molecular dynamics simulations of shear in a biomimetic hydroxyapatite-collagen composite. Our model exhibits elastic properties fully dominated by the inorganic component. However, beyond the elastic regime the biomolecules along with the hierarchical nature of the composite account for the formation of structure-inherent slip zones. These accommodate shear without compromising the overall structure and lead to the sliding of intrinsically defined rods at roughly constant restoring force. Upon releasing load, rod displacement is reversible and backcreep is observed as gradual ionic rearrangement in the slip zone, subjected to an activation barrier.

## Introduction

Hierarchical composites of inorganic crystals and proteins attract increasing interest as biomimetic synthesis routes promise a manifold of new, potentially multi-functional, materials by employing principles of self-organization. Moreover, biomimetic composites also offer the reduction of the complexity inherent to biogenic composites such as teeth and bone. For both experimental characterization and molecular simulation studies, apatite-collagen composites have proven as particularly suited systems which offer the reduction of complexity in the formation conditions but are still able to account for fundamental mechanisms and properties of apatite-based biominerals [1], [2]. On the modeling and simulation side, which shall also be in the focus of the present work, the interplay of ion association to collagen, nucleation of crystalline motifs and mechanisms of self-organization leading to the formation of hierachical composites have been elaborated [3]. Based on this knowledge, scale-up models allow the investigation of the bulk composite and helped to – at least qualitatively – understand aspects of elastic and plastic deformation [4], [5].

The mechanical properties of teeth and bone are unique, as the hardness of apatite is combined with the resilience and self-healing capabilities of biomolecules. While bone and inner parts of teeth (dentine) represent calcified tissues of up to 40 weight-% protein-networks, enamel comprises only 1–2 weight-% of proteins (amelogenins and enamelins). The microstructure of enamel is devised in (mainly) apatite-based prisms that are aligned along the c-axis and arranged as a nano-mosaic [6]. In-between, protein-rich regions facilitate the sliding of prisms with respect to each other, thus allowing enamel to sustain μm indentation without catastrophic failure [7], [8], [9].

For the rationalization of such protein-crystal interface based slip mechanisms at the molecular scale, it is inevitable to take use of simplified models which however must still be able to provide a qualitative account of the underlying phenomena. For simple compression along the c-axis, our recently developed hierarchical apatite-collagen molecular model proved capable of unraveling elastic and plastic deformation mechanisms, including self-healing after releasing mechanical stress [5]. In what follows, we use a considerably larger version of this model to explore shearing and the role of collagen during deformation and relaxation mechanisms.

## Simulation Models and Methods

Based on the molecular model of a single collagen molecule embedded in single-crystalline apatite [3], [10], periodic replication is used to model a hexagonal pattern of 9 explicit collagen molecules embedded into 30×30×10 unit cells of apatite. The simulation system described in ref. [4] was doubled along the a- and b-direction. Refer to ref. [4] for more details of the collagen structure and the interatomic interaction potentials. Periodic boundary conditions are applied to mimic a bulk material which was allowed to relax during constant pressure, constant temperature molecular dynamics simulations mimicking ambient conditions. A time step of 1 fs and Ewald summation with a real space cut-off of 12 Å is used for the molecular dynamics simulations.

**Figure 1:**
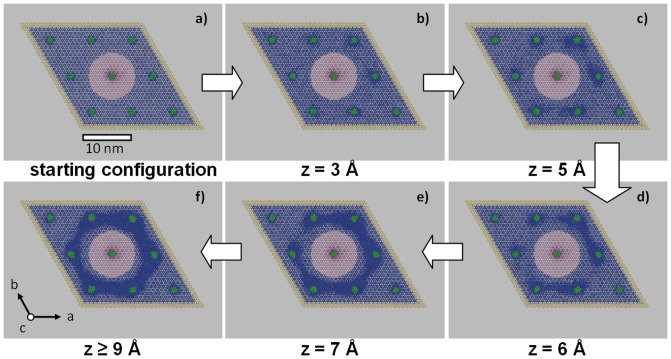
Snapshots of the composite model subjected to periodic boundary conditions in all directions, highlighting molecular dynamics simulations of rod shearing at elevated temperature. For clarity, only the calcium ions and the collagen molecules (green) are shown. Calcium ions are colored in yellow, red and blue to illustrate full position constrains, z-coordinate constrains and unconstraint ions, respectively. Displacement of the central cylinder leads to elastic deformation up to about 3 Å. Plastic deformation is then observed as a local phenomenon starting at the protein-crystal interface (5 Å). The lower row shows the gradual merging of the disordered regions until a ‘complete’ slip zone is formed (9 Å) which embeds the displaced cylinder and facilitates further shearing. Note that the slip zone does not reflect the surface of the cylinder subjected to displacement, but follows the hexagonal pattern of the collagen matrix.

To impose shearing along the c-axis, two sets of constrains were implemented: the outer boundaries of the simulation cell were fixed (constraining all coordinates), whilst in the center of the simulation cell a cylindrical rod of 5 Å radius is subjected to constrains on the z-coordinates only. See figure 1a for an illustration of the model system. Shear is applied by shifting the cylindric rod in steps of 0.01 Å and then allowing the overall model to relax during constant volume, constant temperature molecular dynamics simulations and applying the position/z-coordinate constrains described above. As the model system comprises ∼500.0000 atoms, computational limitations call for fast shearing and testing of rate-dependence is required. For this a series of relaxation times were explored up to 10 ps, leading to consistent results for relaxation periods larger or equal to 1 ps (which corresponds to a shear velocity of 1 m/s). To be on the safe side, the mechanistic studies described in the following were derived from 2 ps relaxation after each 0.01 Å displacement step.

## Results

**Figure 2:**
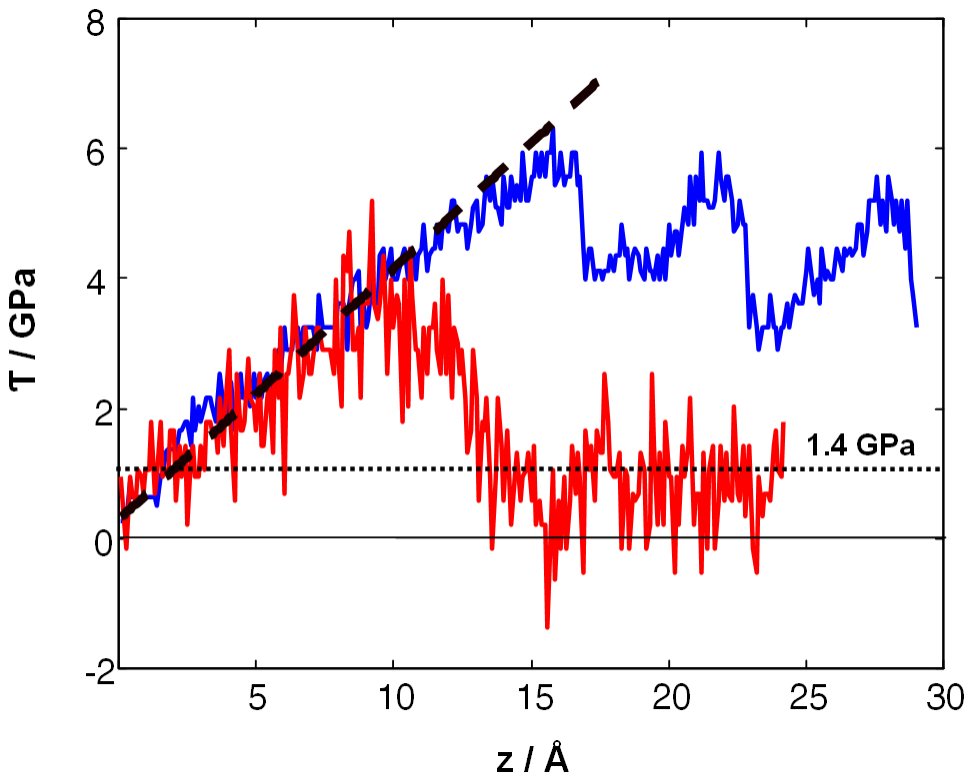
Shear stress-displacement profile for the composite model at 300 K (blue curve) and 1000 K (red curve). At high simulation temperature and displacement by about 10 Å, the single-crystalline model is observed to develop a less-ordered slip zone that facilitates further shearing at roughly constant opposing force leading to a flow stress of 1.4 GPa. The long-dashed curve indicates the elastic region (1.04 GPa / Å×z) whereas the dotted line refers to the average stress observed for the viscous flow regime.

The composite model as illustrated in figure 1a was subjected to shear by displacing the central cylinder parallel to the c-axis of the apatite crystal. This initial molecular dynamics run was performed at 300 K, mimicking ambient conditions. Using displacement rates of 1–5 m/s we observed a consistent shear stress-displacement diagram which indicates elastic deformation up to about 15 Å (fig.2, blue curve). Stress is then partially released by a ∼7 Å slip over the cylinder surface, corresponding to the shifting by a whole c vector of the apatite unit cell. Upon further shearing, this shifting is repeated every 7 Å. Strikingly, each slip event leads to an increase of ionic disordering at the protein-crystal interface. These defects accumulate and lead to a gradually decreasing trend in the shear stress. This hints at a yet un-identified shear mechanism which appears to require considerably longer molecular dynamics runs. While our simulations were consistent for displacement rates within 1–10 m/s, we still expect model relaxation as insufficient to observe mechanisms other than the ‘brute-force’ slipping along the cylinder surface.

The 1–10 m/s rates accessible to direct molecular dynamics simulations correspond to fast loading of biomaterials happening during accidents. However, much smaller rates (fortunately) apply to everyday loading and unloading of teeth and bone. Similar to the accumulation of more and more defects upon increasing shear as discussed above, also slow displacement is expected to be facilitated by reorganization events in the slip zone. While a direct molecular dynamics simulation of slip zone rearrangement starting from the pristine composite model is beyond the scope of computational limitations, we can still prepare simulation models that cover the limiting case of *full relaxation*.

To boost full slip zone optimization during shear at reasonable computational costs, we therefore performed the analogous shearing simulations at elevated temperature. Indeed at 1000 K, still far below the melting temperature (note that also protein unfolding by heat is fully suppressed by the embedding crystal), the mechanistic picture changed drastically. Figure 1b to 1c illustrates the gradual increase of ionic disordering next to the proteins under moderate shearing conditions. This observation is in good analogy to the inset of plasticity under full simulation cell compression along the c-axis as reported in ref. [5]. The key finding of the present study is shown at the lower row of figure 1 (d–f). Upon further displacement of the cylinder in the model center, the disordered regions near the collagen molecules tend to merge and thus establish a less-ordered region that encompasses the cylinder subjected to displacement along the z-coordinate. This disordering of ionic positions is best described as zig-zag and other local displacements and does not refer to melting (which occurs at much higher simulation temperatures). As a consequence of the hexagonal pattern of the collagen proteins inherent to the composite, the region of disordering adopts the shape of a hexagonal prism. Once completed, this hexagonal prism embeds the displaced cylinder and facilitates further shearing. This is reflected by a sharp drop in the shear stress-displacement diagram as shown in figure 2 (red curve). Moreover, further shearing is now found at roughly constant force, indicating a change in mechanism from elastic deformation and slip by a full c-vector length to viscous flow. Repeating the shearing simulations from z = 15 Å, that is after the formation of a hexagonal prism embedded by a less-ordered region, this mechanism was found to remains unchanged when reducing temperature back to 300 K.

**Figure 3:**
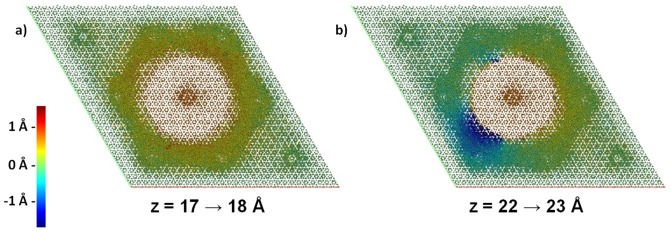
Color-code illustration of the inhomogeneous ionic displacements upon shearing the central rod along the z-axis. Two typical snapshots are shown for a) quasi-elastic shearing with strain propagation into the slip zone, and b) inhomogeneous relaxation within the slip zone. Note that the slip zone may accommodate upwards and downwards displacements at the same time. For clarity, only calcium ions are shown.

It is noteworthy that the slip zone follows the hexagonal pattern of the collagen matrix and does not reflect the surface of the cylinder subjected to enforced displacement. The overall area of the shear interface is thus increased as compared to slipping over the cylinder surface. Nevertheless, a lower shear force results from changing from a single-crystalline to a less-ordered (and comparably protein-rich) slip zone. To illustrate the shear mechanism, it is useful to assign a color code for ionic displacement along the z-axis upon shifting the central rod by 1 Å. We observed two typical scenarios which are roughly equally frequented. In both cases the crystalline regions behave as entities and are either displaced by Δz = 1 Å or remain at their positions. Instead, strain is almost fully localized to the less-ordered slip zone. Therein, ionic displacements may occur roughly homogenous akin to quasi-elastic shearing (fig.3a), but an equally often encountered scenario is that of inhomogeneous relaxation within the slip zone (fig. 3b). Note that in the latter case, the slip zone is observed to accommodate upwards and downwards displacements (ranging from −2 to 2 Å) at the same time. We conclude that it is the combination of both shearing mechanisms that leads to a roughly constant shear force once the slip zone is established (fig.2).

**Figure 4:**
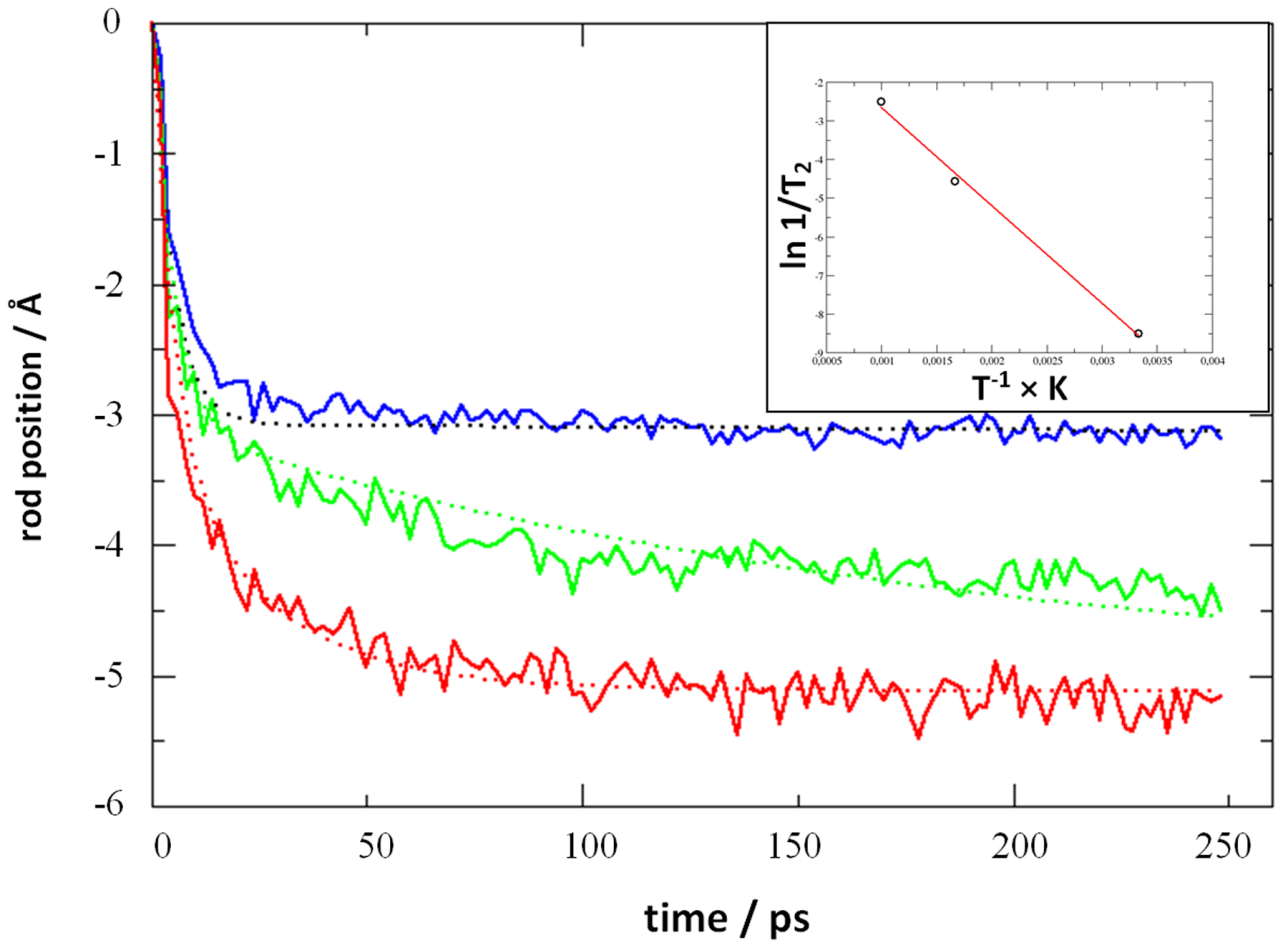
Relaxation after rod displacement by 30 Å as observed from unconstraint molecular dynamics simulations at 300, 600 and 1000 K shown in blue, green and red, respectively. The straight curves indicate double exponential fits with relaxation times of τ_1_ = 4.8 ps and τ_2_ = 9852, 192 and 24.6 ps, respectively. While the temperature-dependent picosecond-scale relaxation reflects elastic response, backcreep is related to an activation energy which was assessed as 21 kJ/mol from an Arrhenius plot (upper right).

In analogy to this dualism of shearing mechanisms accounting for viscous flow, it is intuitive to also expect two disjunct *relaxation processes* when releasing the position constrains of the central rod. Indeed, monitoring the z-position of the central cylinder during relaxation from 30 Å displacement, we find a double-exponential kinetics based on time scales that are separated by three orders of magnitude. It is intuitive to relate rod shifting on the picosecond scale to a viscoelastic relaxation, i.e. a process that does not involve the crossing of energy barriers. On the other hand, non-elastic relaxation such as slip-zone rearrangements occur at much slower pace [7], [8], [9]. Unlike elastic relaxation, such “backcreep” should thus involve an activation barrier. The latter is accessible to molecular dynamics simulations, and, from an Arrhenius plot based on T = 300, 600 and 1000 K, the energy barrier to slip zone reorganization was identified as 21 kJ/mol which is equivalent to 8.4 k_B_T at room temperature (note that the protein is embedded in apatite and does not show temperature-induced denaturation within the nanosecond scale of our simulations). The overall fit for all three relaxation experiments reads:
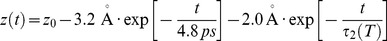
where the constant z_0_ is set to zero in figure 4, the second term refers to temperature-independent elastic relaxation, and the last term describes backcreep.

For assessing the shearing interface area of our molecular model, the mantle surface (A = 6×8.89×6.96 nm^2^) of an ideal hexagonal prism connecting the collagen molecules is assumed as a reasonable approximate. On this basis, the activation barrier to backcreep divided by shearing interface area is estimated as 0.057 kJmol^−1^nm^−2^.

## Conclusion

We demonstrated the use of molecular dynamics simulations for rationalizing complex mechanical processes within a biomimetic hydroxyapatite-collagen composite model. Despite unavoidable simplifications as compared to biogenic materials, it is still possible to at least qualitatively relate our findings to enamel which exhibits many structural similarities and also similar protein content. In both cases, shearing is facilitated by structure-inherent slip zones. These hexagonal zones of reduced ionic ordering and comparably larger protein content accommodate shear strain for the sake of undistorted crystalline regions. The region of reduced ionic ordering establishes an interface between single crystalline regions and allow hexagonal rods to undergo viscous flow - despite the in principle brittle nature of apatite. Elastic relaxation and backcreep mechanisms appear to occur on different time- and length scales, depending on the nature of mechanical load. In an earlier simulation study we found bulk composite compression to involve ionic disordering and reorganization at individual collagen molecules [5]. While this represents a nm-scale phenomenon, in the present work we demonstrate inhomogeneous load applied by shearing to involve 10 nm-scale hexagonal rods. Despite different length scale, also the boundaries of the hexagonal rods are defined by the organic part of the composite.

## References

[1] Kniep R, Simon P (2006) Fluorapatite-Gelatine-Nanocomposites: Self-Organized Morphogenesis, Real Structure and Relations to Natural Hard Materials. Top. Curr. Chem. 270: 73–125.

[2] Zahn D, Hochrein O, Kawska A, Brickmann B, Kniep R (2007) Towards an Atomistic Understanding of Apatite-Collagen Biomaterials: Linking Molecular Simulation Studies of Complex-, Crystal- and Composite-Formation to Experimental Findings. J. Mater. Sci. 42: 8966–8973.

[3] Kawska A, Hochrein O, Brickmann J, Kniep R, Zahn D (2008) On the Nucleation Mechanism of Fluorapatite-Collagen Composites: Ion Association and Motif Control by Collagen Proteins. Angew. Chem. Int. Ed. 120: 5060–5063. 10.1002/anie.200800908 18496823

[4] Duchstein P, Zahn D (2011) Atomistic Modeling of Apatite-Collagen Composites from Molecular Dynamics Simulations extended to Hyperspace. J. Mol. Model. 17: 73–79. 10.1007/s00894-010-0707-7 20372952

[5] Zahn D (2010) A Molecular Rationale of Shock Absorption and Self-Healing in a Biomimetic Apatite-Collagen Composite under Mechanical Load. Angew. Chem. Int.Ed. 49: 9405–9407. 10.1002/anie.201002663 20981745

[6] Maas MC, Dumont ER (1999) Built to last: The structure, function, and evolution of primate dental enamel. Evolutionary Anthropology: Issues, News, and Reviews 8: 133–152.

[7] He LH, Swain MV (2007) Enamel — a 'metallic-like' deformable biocomposite. J. Dentistry 35: 431–437. 10.1016/j.jdent.2006.12.002 17270335

[8] He LH, Swain MV (2007) Contact induced deformation of enamel. Appl. Phys. Lett. 90: 171916–4.

[9] He LH, Swain MV (2008) Nanoindentation creep behavior of human enamel. J. Biomed. Mater. Res. Part A 91: 352–359. 10.1002/jbm.a.32223 18980191

[10] Tlatlik H, Simon P, Kawska A, Zahn D, Kniep R (2006) Biomimetic Fluorapatite-Gelatin Nanocomposites: Structure-Controlling Pretreatment of Gelatin Matrices by Ion Impregnation and its Implications on the Composite Morphology. Angew. Chem. Int. Ed. 45: 1905–1910. 10.1002/anie.200503610 16493717

